# Fungal sensitization and its relationship to mepolizumab response in patients with severe eosinophilic asthma

**DOI:** 10.1111/cea.13680

**Published:** 2020-06-25

**Authors:** Andrew Wardlaw, Peter H. Howarth, Elliot Israel, Camille Taillé, Santiago Quirce, Stephen Mallett, Stewart Bates, Frank C. Albers, Namhee Kwon

**Affiliations:** ^1^ Institute for Lung Health University of Leicester Leicester UK; ^2^ Global Medical Franchise GSK House Brentford UK; ^3^ Clinical and Experimental Sciences Faculty of Medicine University of Southampton and NIHR Respiratory Biomedical Research Unit Southampton General Hospital Southampton UK; ^4^ Respiratory Medical Franchise GSK Brentford UK; ^5^ Harvard Medical School and Brigham and Women's Hospital Boston MA USA; ^6^ AP‐HP, Hôpital Bichat, Service de Pneumologie et Centre de Référence des Maladies Pulmonaires Rares, Dépt Hospitalo‐Universitaire FIRE Université Paris Diderot, INSERM UMR Paris France; ^7^ Department of Allergy Hospital La Paz Institute for Health Research (IdiPAZ), and CIBER of Respiratory Diseases (CIBERES) Madrid Spain; ^8^ Clinical Statistics GSK Uxbridge UK; ^9^ Respiratory Discovery Medicine GSK Stevenage UK; ^10^ Respiratory Medical Franchise GSK Research Triangle Park NC USA; ^11^ Present address: Avillion US, Inc. Northbrook IL USA

To the Editor,

In asthma, sensitization to fungal, perennial or seasonal allergens increases the risk of uncontrolled symptoms, exacerbations and poor disease outcomes.^1^ In severe asthma, typically 20%‐29% of patients show sensitization to ≥1 fungal allergen, with *Aspergillus* being one of the most common.^2^, ^3^, ^4^ These patients have worse lung function, increased risk of oral corticosteroid use, hospitalization and a greater degree of airflow obstruction than patients non‐sensitized to fungal allergens.^3^, ^4^, ^5^

Severe eosinophilic asthma is characterized by frequent exacerbations and elevated eosinophil counts. Currently, there is limited information on the prevalence of fungal allergen sensitization in patients with severe eosinophilic asthma, and its impact on clinical responses to treatments such as the anti‐interleukin (IL)‐5 monoclonal antibody mepolizumab. In clinical trials, mepolizumab reduced exacerbation frequency and oral corticosteroid use, improved lung function, and health‐related quality of life (HRQoL) and symptoms vs placebo in patients with severe eosinophilic asthma.^6^, ^7^ This post hoc analysis of the MENSA study describes the prevalence of fungal sensitization in enrolled patients and their clinical response to mepolizumab.

MENSA was a randomized, double‐blind, Phase III trial (GSK ID: 115588; NCT01691521) in patients with severe eosinophilic asthma.^7^ Patients were randomized (1:1:1) to receive mepolizumab 75 mg intravenously or 100 mg subcutaneously (SC), or placebo, every 4 weeks for 32 weeks plus standard of care (further details in Appendix S1). In this analysis, all treatment groups were pooled and patients were stratified into subgroups based on their sensitization to fungal and/or perennial/seasonal allergens for the analysis of baseline characteristics and all end‐points. Patients were also stratified for selected end‐points based on their fungal allergen combined specific immunoglobulin (Ig)‐E level percentile (0‐≤50th, >50th‐≤75th, 75‐≤90th or >90th percentile) and IgE‐sensitivity to *Aspergillus fumigatus* and/or *Penicillium chrysogenum* (selected because these thermotolerant filamentous fungi are known to colonize the airways and are associated with lung damage in severe asthma),^5^ other fungal or no fungal sensitization. Further information on fungal allergens tested is included in Table S1. Allergen sensitization was defined as serum IgE level ≥0.35 kU/L.

End‐points assessed included the prevalence of fungal and/or perennial/seasonal allergen sensitization and response to mepolizumab at Week 32. Mepolizumab response was determined according to the annual rate of clinically significant exacerbations (see Appendix S1); change from baseline in pre‐bronchodilator forced expiratory volume in 1 second (FEV_1_), St George's Respiratory Questionnaire (SGRQ) score, Asthma Control Questionnaire (ACQ‐5) score and change from baseline in blood eosinophil count. Changes from baseline in eosinophil granule proteins were assessed in patients receiving mepolizumab 100 mg SC or placebo. Descriptive statistical analyses were performed and are described in Appendix S1.

Of the 576 patients included in MENSA, 349 (61%) were sensitized to allergens (fungal [191/576; 33%], perennial [265/576; 46%] or seasonal [166/576; 29%]; Tables S1‐S3). The most common fungal allergens associated with sensitization were *Candida albicans*, *A fumigatus, Malassezia species* and *P chrysogenum*. In particular, 84/576 (15%) patients were sensitized to *A fumigatus* and 58/576 (10%) to *P chrysogenum*. Overall, 51/576 (9%) patients were sensitized to fungal allergens only, 167/576 (29%) to perennial/seasonal allergens only and 131/576 (23%) to both fungal and perennial/seasonal allergens; 198/576 (34%) patients were not sensitized to allergens. Exacerbation history, baseline SGRQ and ACQ‐5 scores, and blood eosinophil counts were similar across all allergen sensitivity groups (Table 1). As expected, total serum IgE levels were higher in patients sensitized to either fungal or non‐fungal allergens vs those without sensitization and were highest in patients sensitized to both (Table 1).

**Table 1:** Patient demographics and baseline characteristics stratified by fungal and perennial/seasonal allergen sensitization

Allergen group	Allergen sensitization group^a^			
	None (N = 198)	Fungal only (N = 51)	Perennial/seasonal only (N = 167)	Fungal and perennial/seasonal (N = 131)
Age, years, mean (SD)	53.2 (13.2)	56.8 (13.0)	46.4 (14.4)	46.9 (14.9)
Female, n (%)	117 (59)	30 (59)	102 (61)	62 (47)
Asthma duration, years, mean (SD)	16.9 (13.0)	21.4 (13.4)	22.0 (13.9)	21.7 (14.6)
Maintenance OCS use, n (%)	62 (31)	17 (33)	49 (29)	34 (26)
Daily OCS dose, mg/d, mean (SD)^b^	12.1 (9.3)	9.3 (7.7)	13.1 (9.6)	13.3 (12.1)
Number of exacerbations in prior year, mean (SD)	3.9 (2.9)	3.5 (1.9)	3.5 (2.7)	3.5 (2.3)
Pre‐BD FEV_1_, L, mean (SD)	1.732 (0.653)	1.593 (0.542)	1.851 (0.668)	1.962 (0.669)
Post‐BD FEV_1_, L, mean (SD)	1.991 (0.664)	1.835 (0.605)	2.191 (0.719)	2.274 (0.731)
Pre‐BD % predicted FEV_1_, mean (SD)	60.8 (18.7)	57.2 (14.6)	60.9 (19.1)	63.0 (17.3)
% reversibility (screening), mean (SD)	27.3 (21.4)	26.4 (16.9)	28.6 (22.8)	28.1 (24.6)
SGRQ total score, mean (SD)	47.1 (18.8)	47.7 (17.7)	45.8 (20.8)	45.5 (20.3)
ACQ‐5 score, mean (SD)	2.1 (1.1)	2.1 (1.2)	2.3 (1.3)	2.2 (1.1)
Blood eosinophil count, geo mean (SD log_e_) cells/μL	310 (1.017)	290 (1.144)	290 (0.975)	300 (0.861)
Total serum IgE, geo mean (SD log_e_) kU/L	63.21 (1.32)	274.51 (1.10)	154.41 (1.24)	533.50 (1.22)

Note

For further information on the allergens included in each group, please refer to the Appendix S1. Data shown in this table are descriptive, and observed differences between groups were not subjected to statistical testing.

Abbreviations: ACQ‐5, Asthma Control Questionnaire; BD, bronchodilator; FEV_1_, forced expiratory volume in 1 s; IgE, immunoglobulin‐E; OCS, oral corticosteroid; SD, standard deviation; SGRQ, St George's Respiratory Questionnaire.

^a^ Twenty‐nine patients did not have allergen sensitization data.

^b^ n = 55, 14, 32 and 24 for respective groups.

After 32 weeks of mepolizumab treatment, annual rates of clinically significant exacerbations were reduced by 48%‐62% vs placebo across the fungal and/or perennial/seasonal allergen sensitization groups (Figure 1A; Table S4). There was no clear trend in exacerbation reduction with increasing baseline combined IgE level to fungal allergens (Table S5). A trend for reductions in the annual rate of clinically significant exacerbations with mepolizumab vs placebo was observed in patients sensitized to *Aspergillus* and/or *Penicillium* (70%) and those not sensitized to fungal allergens (52%) (Figure 1B; Table S4). Although a numerical reduction in exacerbation rate was also observed with mepolizumab vs placebo in patients sensitized to other fungal allergens (44%), this was not as pronounced (Figure 1B).

**FIGURE 1:**
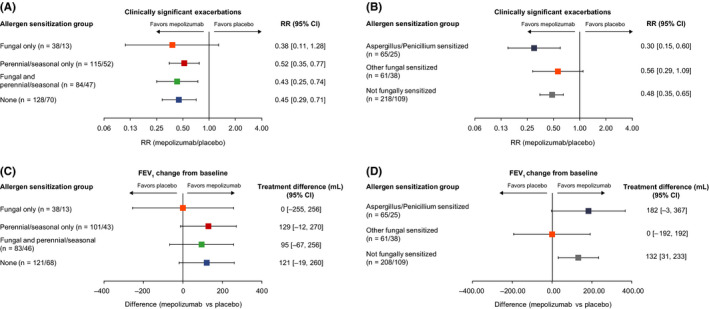
Clinical responses to mepolizumab in patients stratified by fungal and/or perennial/seasonal allergen sensitization and fungal species. “n” represents the number of patients (mepolizumab/placebo) for whom relevant subgroup data were available; clinically significant exacerbations were defined as asthma worsening requiring systemic corticosteroid (intravenously or orally for ≥3 d, or single intramuscular dose), or ER visit or hospitalization; CI, confidence interval; ER, emergency room; FEV_1_, forced expiratory volume in 1 s; RR, rate ratio

Mepolizumab vs placebo was associated with a numerical trend for improved pre‐bronchodilator FEV_1_ from baseline in all patients except those with fungal sensitization only, although this may be due to the small sample size (n = 51) for this group (Figure 1C; Table S4). There was also a trend for improvement in FEV_1_ from baseline with mepolizumab vs placebo in patients sensitized to *Aspergillus and/or Penicillium*, but no treatment difference in patients sensitized to other fungal allergens (Figure 1D; Table S4). SGRQ and ACQ‐5 scores also showed a trend for improvement with mepolizumab vs placebo in all groups (Figure S1; Table S4); the improvement from baseline with mepolizumab exceeded the minimum clinically important difference (MCID) of 4‐points for SGRQ total score and 0.5‐points for ACQ‐5 score in all groups (Table S4).^8^, ^9^ In addition, mepolizumab vs placebo reduced blood eosinophil counts from baseline by 80%‐87% and reduced eosinophil cationic protein and eosinophil‐derived neurotoxin levels in all groups (Table S6).

Overall, these results suggest that approximately two‐thirds of patients with severe eosinophilic asthma are sensitized to allergens and one‐third to fungal allergens. In patients with fungal and perennial/seasonal allergen or only perennial/seasonal allergen sensitization, mepolizumab reduced exacerbation frequency, with a trend for improved HRQoL and disease control, as measured by SGRQ and ACQ‐5 score, respectively, vs placebo. The results were inconclusive in the fungal only subgroup, likely due to the small sample size for this group. In patients sensitized to *Aspergillus* and/or *Penicillium* but not to other fungal allergens, a trend for greater improvements in lung function and the rate of clinically significant exacerbations with mepolizumab vs placebo were seen, supporting the concept of allergic fungal airways disease as a distinct phenotype of asthma.^10^ However, it is worth noting the relatively small sample size (n = 65) of this subgroup, the limited number of aeroallergens tested and that the analyses have to be interpreted with caution owing to the nature of this post hoc analysis.

In conclusion, patients with severe eosinophilic asthma are likely to benefit from mepolizumab treatment. Based on the results from our analysis of those with IgE‐sensitization, individuals sensitized to *Aspergillus* and/or *Penicillium* may demonstrate the greatest response, although further investigation of this effect is required.

## CONFLICT OF INTEREST

PHH, SM, SB and NK are employees of GSK and hold stocks/shares in GSK; FCA was an employee of GSK at the time of this analysis and is now a current employee of Avillion US Inc; AW reports consultancy fees for advisory boards from GSK and Pulmocide, and participation in clinical trials sponsored by AstraZeneca; EI has served as a consultant to and received personal fees from 4D Pharma, AstraZeneca, Bird Rock Bio, Entrinsic Health Solutions, Equillium, Genentech, GSK, Merck, Novartis, Nuvelution Pharmaceuticals, Pneuma Respiratory, Regeneron Pharmaceuticals, Sanofi Genzyme, Sienna Biopharmaceuticals, Teva and Vitaeris Inc; and reports non‐financial support from Boehringer Ingelheim, Circassia, Genentech, GSK, Merck, Teva and Vifor‐Pharma; and other from Vorso Corp; and has received clinical research grants from AstraZeneca, Boehringer Ingelheim, Genentech, GSK, Merck, Novartis, Sanofi, Teva and Vifor‐Pharma; CT declares consultancy services, speaking at conferences and participation in clinical research projects with AstraZeneca, GSK, Novartis, Sanofi and TEVA; SQ has served as a consultant to ALK, AstraZeneca, Boehringer Ingelheim, GSK, Mundipharma, Novartis, Sanofi and Teva and has received lecture fees from AstraZeneca, Chiesi, GSK, Leti, Mundipharma and Novartis.

## FUNDING INFORMATION

This post hoc analysis and the parent study (MENSA; GSK ID: 115588; ClinicalTrials.gov ID: NCT01691521) were funded by GlaxoSmithKline (GSK).

## Supporting information

App S1

## Data Availability

Anonymized individual participant data from the study listed within this publication and their associated documents can be requested for further research from www.clinicalstudydatarequest.com.

## References

[1] GINA . Global strategy for asthma management and prevention June 2019. https://ginasthma.org/wp‐content/uploads/2019/06/GINA‐2019‐main‐report‐June‐2019‐wms.pdf. Accessed January, 2020.

[2] Masaki K , Fukunaga K , Matsusaka M , et al. Characteristics of severe asthma with fungal sensitization. Ann Allergy Asthma Immunol. 2017;119:253‐257. 28801088 10.1016/j.anai.2017.07.008

[3] Goh KJ , Yii ACA , Lapperre TS , et al. Sensitization to Aspergillus species is associated with frequent exacerbations in severe asthma. J Asthma Allergy. 2017;10:131‐140. 28461762 10.2147/JAA.S130459 PMC5407445

[4] Denning DW , O'Driscoll BR , Hogaboam CM , et al. The link between fungi and severe asthma: a summary of the evidence. Eur Respir J. 2006;27:615‐626. 16507864 10.1183/09031936.06.00074705

[5] Woolnough KF , Richardson M , Newby C , et al. The relationship between biomarkers of fungal allergy and lung damage in asthma. Clin Exp Allergy. 2017;47:48‐56. 27805757 10.1111/cea.12848

[6] Chupp GL , Bradford ES , Albers FC , et al. Efficacy of mepolizumab add‐on therapy on health‐related quality of life and markers of asthma control in severe eosinophilic asthma (MUSCA): a randomised, double‐blind, placebo‐controlled, parallel‐group, multicentre, phase 3b trial. Lancet Respir Med. 2017;5:390‐400. 28395936 10.1016/S2213-2600(17)30125-X

[7] GSK . Nucala. Prescribing information. September 2019. https://www.gsksource.com/pharma/content/dam/GlaxoSmithKline/US/en/Prescribing_Information/Nucala/pdf/NUCALA‐PI‐PIL.PDF. Accessed January, 2020.

[8] Jones PW . Interpreting thresholds for a clinically significant change in health status in asthma and COPD. Eur Respir J. 2002;19:398‐404. 11936514 10.1183/09031936.02.00063702

[9] Cloutier MM , Schatz M , Castro M , et al. Asthma outcomes: composite scores of asthma control. J Allergy Clin Immunol Pract. 2012;129:S24‐S33. 10.1016/j.jaci.2011.12.980 PMC4269334 22386507

[10] Woolnough K , Fairs A , Pashley CH , et al. Allergic fungal airway disease: pathophysiologic and diagnostic considerations. Curr Opin Pulm Med. 2015;21:39‐47. 25415407 10.1097/MCP.0000000000000129

