# Supplementary material for: Fungal sensitization and its relationship to mepolizumab response in patients with severe eosinophilic asthma
Source: Clin Exp Allergy. 2020 Jun 25;50(7):869–72. doi: 10.1111/cea.13680 (PMC7540511; doi:10.1111/cea.13680)
Supplement: Supplementary file 1 — App S1 [file CEA-50-869-s001.docx]

# Appendix S1

**MENSA study details**

The MENSA study included patients ≥12 years of age, with ≥2 exacerbations in the prior year requiring systemic corticosteroids despite treatment with high-dose inhaled corticosteroids and additional controller medication(s), with a blood eosinophil count of ≥150 cells/µL at screening or ≥300 cells/µL in the prior year, evidence of airflow obstruction,[^7^](#_ENREF_7) and without bronchopulmonary aspergillosis.

**Definition of clinically significant exacerbations**

Clinically significant exacerbations were defined as asthma worsening requiring systemic corticosteroids, emergency room visits or hospitalization.

**Biomarker measurements**

Levels of the biomarkers, eosinophil cationic protein, eosinophil-derived neurotoxin and IgE were quantified using serum samples obtained at randomization (Week 0) and the exit visit (Week 32).

**Statistical analyses**

Exacerbation endpoints were analyzed with a negative binomial regression model, with covariates of treatment group, baseline maintenance oral corticosteroid (OCS) therapy (OCS vs. no OCS), region, exacerbations in the year prior to the study (as an ordinal variable) and baseline % predicted forced expiratory volume in 1 second (FEV_1_), with time of treatment as an offset variable.

FEV_1_ was analyzed with a mixed model repeated measures model with covariates of baseline, region, baseline maintenance OCS therapy (OCS vs no OCS), exacerbations in the year prior to the study (as an ordinal variable), treatment and visit, plus interaction terms for visit by baseline and visit by treatment group.

St George’s Respiratory Questionnaire (SGRQ) scores were analyzed by analysis of covariance with covariates of baseline, region, baseline maintenance OCS therapy (OCS vs no OCS), exacerbations in the year prior to the study (as an ordinal variable), baseline % predicted FEV_1_, and treatment.

Asthma Control Questionnaire-5 (ACQ-5) scores, blood eosinophil counts, eosinophil granule proteins, and chemokines were analyzed by mixed model repeated measures with covariates of baseline, region, baseline maintenance OCS therapy (OCS vs no OCS), exacerbations in the year prior to the study (as an ordinal variable), baseline % predicted FEV_1_, treatment and visit, plus interaction terms for visit by baseline and visit by treatment group.

**Population sizes**

In total 576 patients were included in MENSA study, but 29 patients did not have allergen data. Therefore, the baseline data and efficacy analyses only included the 547 patients with allergen data available. Details of the missing data are provided **in Supp Tables 1–3.**

**Figure S1.** Change from baseline in SGRQ and ACQ-5 total scores in patients stratified by fungal and/or perennial/seasonal allergen sensitization and fungal species.


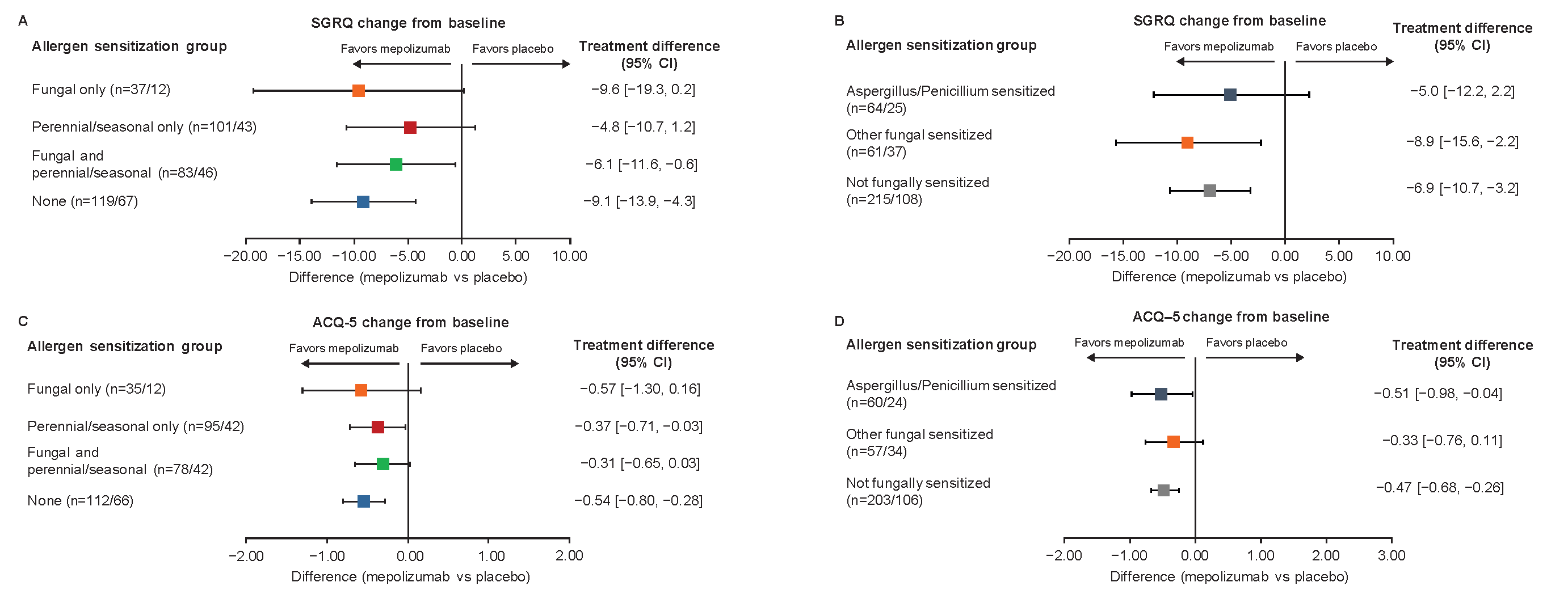


“n” represents the number of patients (mepolizumab/placebo) for whom relevant subgroup data were available.

ACQ-5, Asthma Control Questionnaire; CI, confidence interval; RR, rate ratio; SGRQ, St George’s Respiratory Questionnaire.

**Table S1.** Prevalence of fungal allergen sensitization by fungal species at baseline.

|  | **Sensitization to fungal allergen (N = 576)** | | |
| --- | --- | --- | --- |
| **n (%)** | **Positive** | **Negative** | **Missing** |
| **Any fungal allergen** | 191 (33) | 382 (67) | 3 (<1) |
| **Species** |  |  |  |
| *Candida albicans* | 125 (22) | 409 (71) | 42 (7) |
| *Aspergillus fumigatus* | 84 (15) | 450 (78) | 42 (7) |
| *Malassezia spp.* | 71 (12) | 462 (80) | 43 (7) |
| *Penicillium chrysogenum* | 58 (10) | 476 (83) | 42 (7) |
| *Alternaria alternata* | 53 (9) | 494 (86) | 29 (5) |
| *Fusarium proliferatum* | 47 (8) | 487 (85) | 42 (7) |

Denominator for all proportions is the total population (n = 576); Negative: <0.35 kU/L serum IgE; Positive: ≥0.35 kU/L serum IgE.
IgE, immunoglobulin E.

**Table S2.** Prevalence of perennial allergen sensitization by individual allergen at baseline.

|  | **Sensitization to perennial allergen (N = 576)** | | |
| --- | --- | --- | --- |
| **n (%)** | **Positive** | **Negative** | **Missing** |
| **Any perennial allergen** | 265 (46) | 282 (49) | 29 (5) |
| **Allergen** |  |  |  |
| *Dermatophagoides farinae  (American house dust mite)* | 195 (34) | 351 (61) | 30 (5) |
| *Dermatophagoides pteronyssinus (European house dust mite)* | 188 (33) | 359 (62) | 29 (5) |
| *Dog dander* | 141 (25) | 406 (70) | 29 (5) |
| *Cat dander* | 136 (24) | 411 (71) | 29 (5) |
| *Cockroach* | 80 (14) | 466 (81) | 30 (5) |

Denominator for all proportions is the total population (n = 576); Negative: <0.35 kU/L serum IgE; Positive: ≥0.35 kU/L serum IgE.
IgE, immunoglobulin E.

**Table S3.** Prevalence of seasonal allergen sensitization by individual allergen at baseline.

|  | **Sensitization to seasonal allergens (N = 576)** | | |
| --- | --- | --- | --- |
| **n (%)** | **Positive** | **Negative** | **Missing** |
| **Any seasonal allergen** | 166 (29) | 381 (66) | 29 (5) |
| **Allergen** |  |  |  |
| *Lolium perenne* pollen (wild rye pollen) | 131 (23) | 416 (72) | 29 (5) |
| *Olea europaea* pollen (olive tree pollen) | 76 (13) | 471 (82) | 29 (5) |
| *Quercus alba* pollen (white oak pollen) | 79 (14) | 468 (81) | 29 (5) |
| *Ulmus glabra* pollen (wych elm pollen) | 59 (10) | 488 (85) | 29 (5) |
| *Salsola kali* pollen (Russian thistle) | 44 (8) | 503 (87) | 29 (5) |

Denominator for all proportions is the total population (n = 576); Negative: <0.35 kU/L serum IgE; Positive: ≥0.35 kU/L serum IgE.
IgE, immunoglobulin E.

**Table S4.** Clinical responses to mepolizumab in patients stratified by fungal and/or perennial/seasonal allergen sensitization and fungal species.

|  | **Allergen sensitization group** | | | | | | | | **Fungal sensitization group** | | | | | |
| --- | --- | --- | --- | --- | --- | --- | --- | --- | --- | --- | --- | --- | --- | --- |
| **Allergen sensitization group** | **None** | | **Fungal only** | | **Perennial/seasonal only** | | **Fungal and perennial/ seasonal** | | **Aspergillus/ Penicillium sensitized** | | **Other fungal sensitized** | | **Not fungally sensitized** | |
|  | **Mepo** | **Placebo** | **Mepo** | **Placebo** | **Mepo** | **Placebo** | **Mepo** | **Placebo** | **Mepo** | **Placebo** | **Mepo** | **Placebo** | **Mepo** | **Placebo** |
| **Clinically significant exacerbations, n** | 128 | 70 | 38 | 13 | 115 | 52 | 84 | 47 | 65 | 25 | 61 | 38 | 218 | 109 |
| Exacerbation rate/year | 0.80 | 1.77 | 0.64 | 1.68 | 0.82 | 1.59 | 0.78 | 1.81 | 0.70 | 2.35 | 0.90 | 1.61 | 0.81 | 1.69 |
| RR, mepo/placebo (95% CI) | 0.45 (0.29, 0.71) | | 0.38 (0.11, 1.28) | | 0.52 (0.35, 0.77) | | 0.43 (0.25, 0.74) | | 0.30 (0.15, 0.60) | | 0.56 (0.29, 1.09) | | 0.48 (0.35, 0.65) | |
| **FEV_1_ change from baseline, n** | 121 | 68 | 38 | 13 | 101 | 43 | 83 | 46 | 65 | 25 | 61 | 38 | 218 | 109 |
| LS mean (SE) at Week 32 | 1978 (41.7) | 1858 (56.5) | 1744 (60.3) | 1744 (107.4) | 2045 (39.3) | 1916 (59.1) | 2115 (48.2) | 2020 (64.9) | 2092 (48.0) | 1910 (78.4) | 1927 (59.1) | 1927 (75.2) | 2011 (29.4) | 1879 (41.7) |
| Treatment difference, mL (95% CI) | 121 (-19, 260) | | 0 (-255, 256) | | 129 (-12, 270) | | 95 (-67, 256) | | 182 (-3, 367) | | 0 (-192, 192) | | 132 (31, 233) | |

|  | **Allergen sensitization group** | | | | | | | | **Fungal sensitization group** | | | | | |
| --- | --- | --- | --- | --- | --- | --- | --- | --- | --- | --- | --- | --- | --- | --- |
| **Allergen sensitization group** | **None** | | **Fungal only** | | **Perennial/seasonal only** | | **Fungal and perennial/ seasonal** | | **Aspergillus/ Penicillium sensitized** | | **Other fungal sensitized** | | **Not fungally sensitized** | |
|  | **Mepo** | **Placebo** | **Mepo** | **Placebo** | **Mepo** | **Placebo** | **Mepo** | **Placebo** | **Mepo** | **Placebo** | **Mepo** | **Placebo** | **Mepo** | **Placebo** |
| **SGRQ change from baseline, n** | 119 | 67 | 37 | 12 | 101 | 43 | 83 | 46 | 64 | 25 | 61 | 37 | 215 | 108 |
| LS mean (SE) at Week 32 | 28.8 (1.43) | 37.9 (1.93) | 30.1 (2.22) | 39.7 (4.09) | 32.0 (1.61) | 36.7 (2.50) | 31.8 (1.61) | 37.9 (2.19) | 29.6 (1.83) | 34.6 (3.02) | 32.8 (2.02) | 41.8 (2.62) | 30.3 (1.08) | 37.2 (1.54) |
| LS mean change from baseline (SE) at Week 32 | -18.8 (1.43) | -9.7 (1.93) | -17.1 (2.22) | -7.6 (4.09) | -14.5 (1.61) | -9.8 (2.50) | -13.2 (1.61) | -7.1 (2.19) | -12.8 (1.83) | -7.9 (3.02) | -15.7 (2.02) | -6.8 (2.62) | -16.8 (1.08) | -9.9 (1.54) |
| Treatment difference (95% CI) | -9.1 (-13.9, -4.3) | | -9.6 (-19.3, 0.2) | | -4.8 (-10.7, 1.2) | | -6.1 (-11.6, -0.6) | | -5.0 (-12.2, 2.2) | | -8.9 (-15.6, -2.2) | | -6.9 (-10.7, -3.2) | |
| **ACQ-5 change from baseline, n** | 112 | 66 | 35 | 12 | 95 | 42 | 78 | 42 | 60 | 24 | 57 | 34 | 203 | 106 |
| LS mean (SE) at Week 32 | 1.06 (0.080) | 1.60 (0.105) | 1.12 (0.166) | 1.69 (0.304) | 1.42 (0.095) | 1.79 (0.143) | 1.40 (0.101) | 1.71 (0.138) | 1.25 (0.122) | 1.76 (0.198) | 1.40 (0.129) | 1.72 (0.168) | 1.20 (0.062) | 1.68 (0.087) |
| LS mean change from baseline (SE) at Week 32 | -1.01 (0.080) | -0.47 (0.105) | -0.99 (0.166) | -0.42 (0.304) | -0.87 (0.095) | -0.50 (0.143) | -0.83 (0.101) | -0.52 (0.138) | -0.81 (0.122) | -0.30 (0.198) | -0.99 (0.129) | -0.67 (0.168) | -0.97 (0.062) | -0.50 (0.087) |
| Treatment difference (95% CI) | -0.54 (-0.80, -0.28) | | -0.57 (-1.30, 0.16) | | -0.37 (-0.71, -0.03) | | -0.31 (-0.65, 0.03) | | -0.51 (-0.98, -0.04) | | -0.33 (-0.76, 0.11) | | -0.47 (-0.68, -0.26) | |

CI, confidence interval; LS, least squares; n, number of patients with analyzable data at time point; mepo, mepolizumab; RR, rate ratio; SE, standard error.

**Table S5.** Annual rate of clinically significant exacerbations stratified by baseline fungal allergen combined specific IgE levels.

|  | | **Baseline fungal allergen combined IgE (N = 576)** | | | | | | | | | | |
| --- | --- | --- | --- | --- | --- | --- | --- | --- | --- | --- | --- | --- |
|  |  | **0–≤50^th^ percentile** | | | **>50–≤75^th^ percentile** | | | **>75–≤90^th^ percentile** | | | **>90^th^ percentile** | |
|  | **Mepo** | | **Placebo** | **Mepo** | | **Placebo** | **Mepo** | | **Placebo** | **Mepo** | | **Placebo** |
| **Annual rate of clinically significant exacerbations** | | | |  | |  |  | |  |  | |  |
| n | 192 | | 101 | 96 | | 41 | 58 | | 28 | 37 | | 20 |
| Annual rate | 0.85 | | 1.80 | 0.98 | | 1.24 | 0.57 | | 1.76 | 0.79 | | 1.84 |
| RR mepo/placebo  (95% CI) | 0.47  (0.33, 0.66) | | | 0.79  (0.48, 1.32) | | | 0.32  (0.16, 0.65) | | | 0.43  (0.19, 0.95) | | |

Percentiles are based on the combined IgE level for all fungal allergens assessed. Percentile values – 50^th^: 0.425; 75^th^: 1.265; 90^th^: 4.935 kU/L.
CI, confidence interval; IgE, immunoglobulin E; mepo, mepolizumab; RR, rate ratio.

**Table S6.** Change from baseline in specific biomarkers following mepolizumab treatment stratified by fungal and/or perennial/seasonal allergen sensitization.

|  | **Allergen sensitization group** | | | | | | | |
| --- | --- | --- | --- | --- | --- | --- | --- | --- |
|  | **None** | | **Fungal only** | | **Perennial/seasonal only** | | **Fungal and perennial/seasonal** | |
|  | **Mepo** | **Placebo** | **Mepo** | **Placebo** | **Mepo** | **Placebo** | **Mepo** | **Placebo** |
|  | **N = 125** | **N = 68** | **N = 37** | **N = 13** | **N = 109** | **N = 51** | **N = 84** | **N = 46** |
| **Blood eosinophil counts, n** | 117 | 65 | 37 | 13 | 98 | 40 | 82 | 44 |
| LS mean (SE logs) ratio to baseline at Week 32 | 0.16 (0.09) | 0.78 (0.12) | 0.17 (0.15) | 0.87 (0.27) | 0.16 (0.08) | 0.85 (0.13) | 0.13 (0.10) | 1.00 (0.13) |
| Ratio, mepo/placebo (95% CI) | 0.20 (0.15, 0.27) | | 0.20 (0.11, 0.38) | | 0.18 (0.14, 0.25) | | 0.13 (0.09, 0.18) | |
| **ECP, n** | 60 | 67 | 18 | 13 | 55 | 41 | 41 | 46 |
| LS mean (SE logs) ratio to baseline at Week 32 | 0.37 (0.09) | 0.75 (0.09) | 0.37 (0.17) | 0.80 (0.20) | 0.40 (0.09) | 1.20 (0.10) | 0.44 (0.14) | 0.91 (0.13) |
| Ratio, mepo/placebo (95% CI) | 0.49 (0.38, 0.63) | | 0.47 (0.25, 0.86) | | 0.33 (0.25, 0.44) | | 0.48 (0.32, 0.71) | |
| **EDN, n** | 60 | 63 | 19 | 12 | 54 | 41 | 40 | 45 |
| LS mean (SE logs) ratio to baseline at Week 32 | 0.28 (0.07) | 0.85 (0.07) | 0.24 (0.15) | 0.69 (0.19) | 0.26 (0.09) | 1.04 (0.10) | 0.32 (0.09) | 0.97 (0.08) |
| Ratio, mepo/placebo (95% CI) | 0.33 (0.27, 0.40) | | 0.35 (0.20, 0.60) | | 0.25 (0.19, 0.32) | | 0.34 (0.26, 0.44) | |

CI, confidence interval; ECP, eosinophil cationic protein; EDN, eosinophil-derived neurotoxin; LS, least squares; n, number of patients with analyzable data at time point; mepo, mepolizumab; SE, standard error.
